# Cost-effectiveness of cancer interventions in Rwanda: literature review and expert elicitation for health benefits package design

**DOI:** 10.1136/bmjph-2025-003718

**Published:** 2026-03-05

**Authors:** Cassandra Nemzoff, Andres Madriz-Montero, Inga Mumukunde, Jean Marie Vianney Sindambiwe, Isabelle de Valois Ndishimye, Valentine Uyisabye, James Humuza, Rob Baltussen, Regis Hitimana, Sedona Sweeney, Stella Matutina Umuhoza, Anna Vassall

**Affiliations:** 1 London School of Hygiene and Tropical Medicine, London, UK; 2 Clinton Health Access Initiative, Kigali, Rwanda; 3 University of Rwanda, School of Public Health, Kigali, Rwanda; 4 Radboud University Medical Center, Nijmegen, Netherlands; 5 Rwanda Social Security Board, Kigali, Rwanda

**Keywords:** economics, methods, Public Health

## Abstract

**Introduction:**

Prioritising health benefits packages (HBPs) that specify which health services are covered under insurance is sometimes done in disease-specific clusters. Cancer is a good candidate for this, given its high cost and rising disease burden, particularly in low- and middle-income countries.

The Government of Rwanda assessed 49 cancers against nine criteria to inform the design of its HBP. Each cancer had a basic, core and enhanced package of services, and one preventive intervention was assessed, totalling 148 interventions. This paper focuses on the results of one criterion: cost-effectiveness. The objectives were to specify which cost-effectiveness methods were selected and why; to assess the cost-effectiveness of 148 cancer interventions; and to recommend how to strengthen the global cost-effectiveness evidence base.

**Methods:**

Methods were selected using an adaptive health technology assessment approach, by considering the trade-offs between available time, data and capacity. The assessment undertook a review of the Tufts cost-effectiveness assessment (CEA) registry and filled evidence gaps with structured expert elicitation. Analysts summarised lessons learnt to recommend improvements to the global cost-effectiveness evidence base.

**Results:**

Of the 148 cost-effectiveness ratios (CERs) sought, 39 were from the Tufts registry and 83 were expert elicited. Limited availability of CERs from the literature resulted in a disproportionate number of CERs being elicited from experts. Analysts recommend better reporting and improved consistency in the extraction of CEAs to support HBP design.

**Conclusion:**

This is the first study to assess the cost-effectiveness of many cancers simultaneously for HBP design. It highlights the strengths and limitations of existing evidence and demonstrates the feasibility of combining rapid review with expert elicitation to obtain replicable CER estimates. These cost-effectiveness findings were used to prioritise a package of cancer services for Rwanda alongside several other criteria (reported separately).

**WHAT IS ALREADY KNOWN ON THIS TOPIC**

- Prioritising health benefits packages often includes reviewing existing evidence on cost-effectiveness for a variety of disease areas.

**WHAT THIS STUDY ADDS**

- This study is the first of its type to assess cost-effectiveness for a full package of services for 49 cancers, and the first of its type to report in detail expert elicitation of cost-effectiveness for interventions that do not have existing cost-effectiveness evidence.

**HOW THIS STUDY MIGHT AFFECT RESEARCH, PRACTICE OR POLICY**

- The ranking and evidence for cancer services presented in this paper could be adapted in other low- and middle-income countries to facilitate the design of their own cancer benefits packages.

## Introduction

 Health benefits packages (HBPs) are one of the most common policy instruments used by countries striving to achieve universal health coverage. An explicit HBP defines which health services are paid for, by whom and for which patients.^1^ To ensure HBPs are financially sustainable, there is a need to balance demand for new services with a constrained budget and health system. This is often done through a formal, deliberative priority setting process known as health technology assessment (HTA).^2^

HBPs have been prioritised in many countries, often taking a ‘sectoral’ approach that assesses a broad set of health services.^3^,^5^ An alternative is to focus on disease-specific clusters, which reduces the analytical burden of full HBP design while still addressing a broader set of topics compared with a single intervention. Given the rising global burden of disease and high costs associated with cancer treatment, cancer is a suitable candidate for a disease-specific assessment.^6^ Resources that have been developed to aid in prioritising cancer services for HBPs include Disease Control Priorities, with one of its nine volumes on essential health services in low- and middle-income countries (LMICs) dedicated to cancer and the National Comprehensive Care Network’s (NCCN’s) resource-stratified guidelines, which define cancer treatment pathways based on available resources.^7 8^

The Government of Rwanda, through the Ministry of Health (MoH), recently led a process of prioritising cancer services. This was part of a wider government effort aimed at ensuring the financial sustainability of its community-based health insurance (CBHI) scheme, while responding to demand for coverage of new services. The CBHI covers more than 80% of the population^9^ and is managed by the Rwanda Social Security Board. A multi-stakeholder process of assessment and appraisal was designed to prioritise 49 cancers against nine locally relevant criteria, including: cost-effectiveness; burden of disease; financial risk protection; cost; budget impact; feasibility; vulnerable groups; individual effectiveness; and life-threatening conditions.^10^ This paper focuses on results of the cost-effectiveness assessment, which is often a central criterion in HBP assessments as it enables the ranking of interventions to illustrate the optimal mix of services to maximise population health.^11 12^

Despite cost-effectiveness being a key feature of HBPs since the 1990s, gaps in methodological literature remain.^13^ First, HBP assessments are data-demanding, which necessitates the use of ‘adaptive’ health technology assessment (aHTA) methods. aHTA methods deliberately adjust assessments for local time, data and capacity constraints and leverage data from other jurisdictions where possible, rather than conduct analyses from scratch.^14^ However, why and how these methods are selected is not reported in the priority setting literature. Second, practice reports that include details about how cost-effectiveness ratios (CERs) were sourced, transferred and presented are sparse.^15^ Finally, there are limited recommendations in the literature about how to improve the global cost-effectiveness evidence base to support HBPs.^16^

This paper serves to support Rwanda’s cancer prioritisation and fill the gaps in the literature through three objectives: to specify which cost-effectiveness methods were selected and why; to assess the cost-effectiveness of cancer services and report how transferability was accounted for; and to make recommendations on how to strengthen the global cost-effectiveness evidence base.

## Methods

### Defining the services to be assessed

Two rounds of cancer assessments were undertaken. The first (‘round 1’) piloted the assessment methods by assessing the top seven cancers and top three childhood cancers by incidence according to the Rwanda Cancer Registry (n=10) (online supplemental appendix 1). The subsequent assessment (‘round 2’) grouped and assessed all remaining cancers in the Rwanda National Cancer Treatment Guidelines (n=39).^17^ The full list of cancers assessed is available in online supplemental appendix 2.

To define the cancer services, each cancer was divided into three ‘packages’: basic, core and enhanced. This was based on the NCCN’s ‘resource stratification framework’ which uses the available evidence and global clinicians’ expertise to stratify cancer services based on availability, affordability and cost-effectiveness.^18^ The basic package includes the basic minimal standard of care which improves disease-specific outcomes; core includes basic plus additional care that provides major outcome improvements without being cost-prohibitive; and enhanced includes core plus additional care that provides lesser disease outcomes and is cost-prohibitive.^18^ One additional ‘prevention’ package was added for human papillomavirus (HPV) vaccination for cervical cancer prevention. As part of the broader HBP process, a local expert committee was consulted to refine the packaging of services into these categories for appropriateness in Rwanda (online supplemental appendix 4).

### Methods selection

Our cost-effectiveness assessment (CEA) methods were driven by the available analytical time, data and capacity for assessment. We made use of a recent aHTA framework which explicitly considers these constraints against four possible methods: expert opinion, rapid review, transfer or new model.^19^ Local data were available for costs and coverage for a subset of the cancers, but there was only one Rwanda CEA estimate available.^20^

The local assessment team was composed of two senior researchers and eight research assistants from the University of Rwanda School of Public Health. They were supported by three experienced health economists from the London School of Hygiene and Tropical Medicine and the Center for Global Development, members of the international Decision Support Initiative network.^21^

Given a proposed short time frame, limited local cost-effectiveness data and a small assessment team, it was agreed that transferring existing models or developing new models for the long list of cancers was not feasible. Rather, a rapid review of existing literature was conducted using the Tufts CEA registry as the primary data source for CER estimates.^22^ The Tufts registry is useful for HBPs, because it offers a database of more than 12 000 global cost-effectiveness studies with pre-extracted data including CERs and quality scores. This can save significant analytical time and minimise the need to review original studies, though it does exclude studies that have outcomes other than disability-adjusted life years (DALYs) averted or quality-adjusted life years (QALYs) gained. Where cost-effectiveness studies were not available, it was agreed to fill gaps with structured expert elicitation (SEE).^23^

While patients and the public were involved in the general review of the HBP in Rwanda, they were not involved in the estimation of cost-effectiveness presented in this paper.

### Cost-effectiveness assessment

The full search strategy developed for the Tufts registry can be found in online supplemental appendix 3. In summary, it combined keywords related to cancer in general, keywords for the specific cancers being assessed and drugs used for cancer treatment. Keywords were drawn from the local treatment guidelines and a recent unpublished analysis of cancer drug costs in Rwanda.^17 24^ The searches were run on 2 October 2022 for round 1 and 6 June 2023 for round 2.

### Selection of studies from the literature

The best estimate of cost-effectiveness for each basic, core and enhanced cancer ‘package’ was selected from the literature review by assessing their transferability bias for the local context. For this, the Welte’s ‘knock-out’ criteria were adapted because of their simplicity and common use in HBP assessments.^4 25 26^ The criteria include relevance of the intervention and comparator; geographic relevance; and quality of the study. We included studies with both DALYs averted and QALYs gained and used them interchangeably, aligned with recent findings that this practice is acceptable.^27^ This was useful for economic evaluations of cancer interventions, which disproportionately used QALYs, even though DALYs are more often used in LMIC settings. Studies with irrelevant interventions or comparators, or from high-income countries (HICs) were excluded.

A three-step approach was taken to select cost-effectiveness studies: (1) study selection (divided into parts a, b and c); (2) CER adjustment; and (3) CER scoring. Two members of the assessment team reviewed each paper in step 1, with decisions resolved by consensus. Steps 2 and 3 were completed by the first author.

In step 1a, titles and abstracts were reviewed for general relevance. Studies from HICs were removed. Those which were generally irrelevant were also removed, such as those not focused on cancers being assessed or interventions not provided in Rwanda.

In step 1b, each CER within a study was reviewed for the relevance of the intervention and comparator. We considered whether the intervention matched the package in Rwanda, including screening and treatment.

In step 1c, CERs were selected for each cancer and each package (basic, core and enhanced). This included giving geographic preference to studies from lower-middle-income countries; selecting the best match intervention and comparator for each package if there were many to choose from; and ensuring the final CER selection approach was consistent across cancers. Additionally, we recalculated CERs to ensure that the average cost-effectiveness ratio (ACER) was used by dividing the cost of the intervention by the effect of the intervention (rather than the difference in the cost and effect of the study-specific intervention and comparator). This enabled the consistent ranking of interventions against a null comparator. The extracted data from the Tufts registry was used to recalculate the ACER, and when necessary, this was validated against the original study. In some instances, we recalculated an ACER based on the comparator of the study. For example, if the comparator of the study matched one of our packages, we divided the costs by the effects of that comparator to recalculate the ACER.

In step 2, CERs were adjusted for purchasing power parity (PPP) and scored. We adjusted for PPP to standardise across geographies, using the following formula:


$${\text{CER}}_{\text{Rwanda}}={\text{CER}}_{\text{Country X}}\times \frac{\text{GDP}\;\text{per}\;\text{capita}\;{\text{PPP}}_{\text{Rwanda}}}{{\text{GDP}\;\text{per}\;\text{capita}\;\text{PPP}}_{\text{Country x}}}$$

Then, each CER was assigned between one and three stars, where three stars is the most transferable within a transferability factor and nine is the best possible score, replicating a similar approach conducted in Pakistan.^28^ This was based on three transferability factors: geographic relevance, relevance of the intervention and comparator, and quality (table 1).

In step 3, cost-effectiveness ratios were ranked from lowest to highest and accompanied by the star rating.

**Table 1:** Scoring cost-effectiveness ratios

Measure	Measurement approach	3*	2*	1*
Geographic relevance	Country/income level	Rwanda or other African country	Lower-middle-income country	Upper-middle-income country
Relevance of intervention/comparator	Reviewers’ interpretation	Exact match	Partial match	No match
Quality	Tufts quality scoring framework	4–7	2–4	1 or unscored

### Expert elicitation

To fill gaps where CERs were not available in the literature, we undertook a process of SEE, guided by standard and local approaches to SEE.^23 29^ A group of 12 cancer experts (online supplemental appendix 4), hereafter referred to as ‘the cancer experts’, were nominated by the MoH and formally invited to support the full HBP design process and participated in SEE. They consented to participate in the overall HBP design process as anonymous contributors via face-to-face consultations by responding to the invitation from the MoH.

An expert consensus approach was used for the elicitation of effectiveness and subsequently CERs. First, an estimate of individual effectiveness was elicited for all interventions being assessed. Experts were divided into small groups with a facilitator and were asked to rate cancer packages as low effectiveness (the person would survive for less than 6 months after intervention); medium effectiveness (the person would survive between 6 months and 5 years); or high effectiveness (the person would survive more than 5 years). Each group agreed on a rating by consensus. Second, these estimates were combined with other data from the broader HBP assessment on each package and shared with experts. This included a detailed explanation of each package, a health sector unit cost per case and the CERs that were identified for other packages from the Tufts registry for reference. Third, experts were asked to individually score each service for cost-effectiveness using the descriptions in table 2. Experts then shared their scores within the same group, and the group discussed a consensus score. Finally, all experts were convened and presented with the full list of scores for validation.

**Table 2:** Cost-effectiveness categorisation

Category	Level of cost-effectiveness	Typical characteristics	ICER range
1	Not cost-effective	High costs and low effects	>US$2502 (3× GDP pc)
2	Potentially not cost-effective	High costs and medium/high effects Medium costs and low/medium effects	US$834−US$1668 (1–3× GDP pc)
3	Potentially cost-effective	Medium costs and high effects Low costs and low/medium effects	US$417−US$834 (0.5–1× GDP pc)
4	Very cost-effective	Low costs and high effects	<US$417 (0.5×GDP pc)

GDP pc, gross domestic product per capita; ICER, incremental cost-effectiveness ratio.

The potential range of CERs for each category reflects a rough estimate of thresholds based on gross domestic product per capita (GDP pc). CERs elicited from this process were assigned to each category as follows: 1=US$2502 (3× GDP pc); 2=US$1668 (2× GDP pc); 3=US$834 (1× GDP pc); and 4=US$417 (0.5× GDP pc).^30 31^ These estimates broadly align with recent cost-effective threshold estimates for Rwanda of US$325 to US$426 or 39%–51% of GDP pc (These estimates inflate 2015 estimates to 2022, using the consumer price index (CPI) from the World Bank: https://data.worldbank.org/indicator/fp.cpi.totl?end=2022&locations=RW&start=1966&view=chart).^32^

## Results

### CERs from the literature

Round 1 included packages of treatment for 10 cancers (n=30, where a CER was sought for basic, core and enhanced for each cancer) and prevention for one cancer (HPV vaccination, n=1). Cancers included breast, cervical, gastric, colon, rectal, prostate, liver, Wilms, retinoblastoma and acute lymphoblastic leukaemia. We identified 2481 cancer studies in the Tufts Registry for review. Of these, 124 from LMICs were included in step 1a. These 124 studies contained 1100 CERs, 164 of which were selected in step 1b. In step 1c, a final list of 20 CERs from nine studies was included (n=20/31). Round 2 sought CERs for the remaining 39 cancers (n=117). We identified 1576 studies. Of these, 133 from LMICs were included in step 1a. These 133 studies contained 311 CERs, of which 106 remained in step 1b. Finally, 19 CERs from 16 studies were selected in step 1c (n=19/117). A list of all studies included can be found in online supplemental appendix 5.

**Figure 1:**
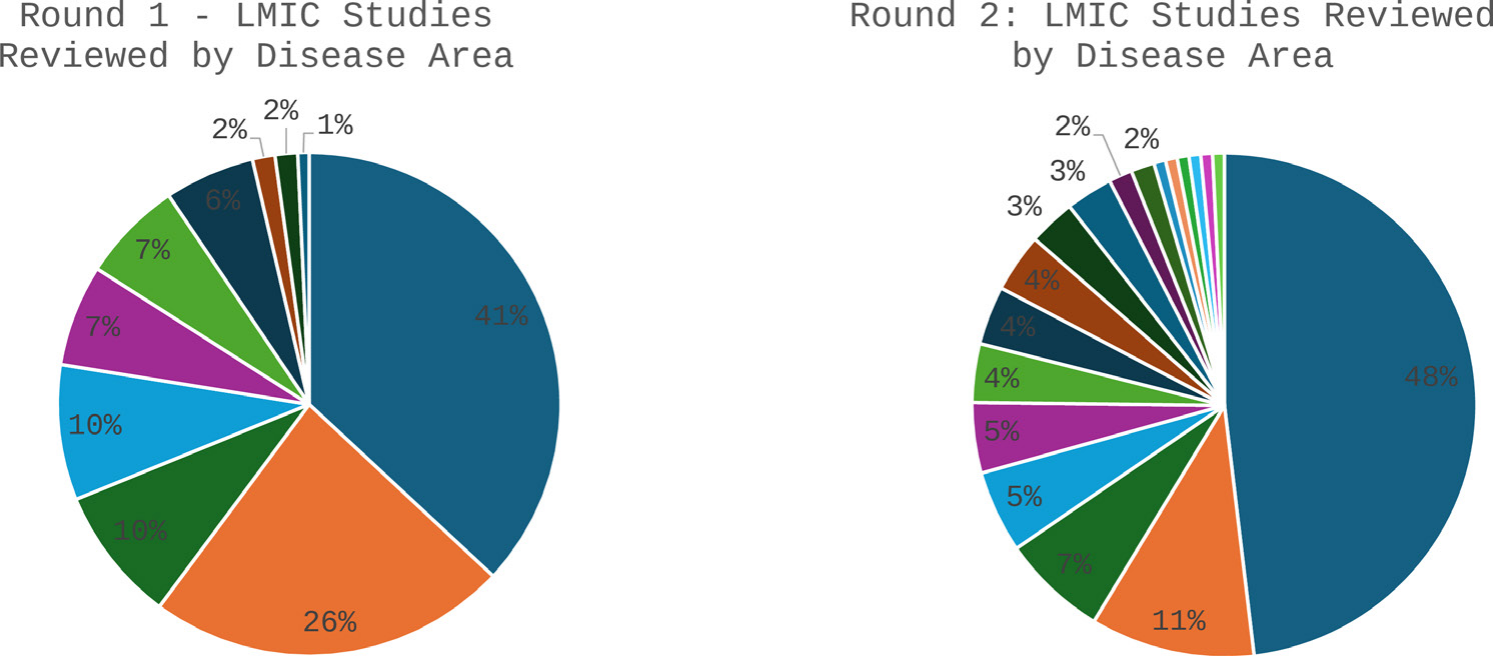
Studies reviewed by disease area. LMIC, low- and middle-income country.

There was a clear publication bias in the studies selected at the end of step 1a in both rounds (figure 1). In round 1, most studies were focused on cervical and breast cancer, with far fewer studies on the remaining eight cancers. In round 2, nearly half of all studies were for non-small cell lung cancer (NSCLC). Moreover, we only found studies for 19 of the 39 cancers in this round, meaning that for the remaining 20 cancers, no cost-effectiveness evidence was available at all. In narrowing the CERs from step 1a to step 1c, exact matches were more likely to be found among the cancers with more studies, and partial matches where there were few to choose from.

The 39 CERs selected are summarised in table 3. Exact matches were found in 20 CERs, and partial matches were found in 19 CERs (A). Geographically, 17 were from lower-middle-income countries and 22 were from upper-middle-income countries (B). The Tufts quality score in the studies ranged from 1 to 7 (C). Quality scores were unavailable for 14 CERs from lower-middle-income countries. All CERs scored two or three stars (D).

**Table 3:** Cost-effectiveness ratios from the literature and transferability scoring

Round	Cancer and level	Cost-effectiveness ratio (2021 USD/DALY)	(A) Intervention and comparator Exact match=3* Partial match=2* No match=1*	(B) Geographic relevance Rwanda or Africa=3* LMIC=2* UMIC=1*	(C) Quality score 4–7=3* 1–4=2* 1 or unscored=1*	(D) Average score (*, **, ***)
1	Cervical—Prevention	212	Exact match	Rwanda		**
1	Gastric—Basic	381	Exact match	UMIC	6.0	**
1	ALL—Basic	432	Partial match	LMIC		**
1	Wilm’s tumour—Basic	445	Partial match	Africa		**
1	Retinoblastoma—Basic	459	Partial match	Africa		**
1	Colon—Core	495	Partial match	Africa		**
1	Rectal—Core	495	Partial match	Africa		**
1	Cervical—Basic	644	Exact match	Africa		**
1	Breast—Core	645	Exact match	Africa		**
1	Colon—Enhanced	650	Exact match	Africa		**
1	Rectal—Enhanced	650	Exact match	Africa		**
1	Cervical—Enhanced	655	Exact match	Africa		**
1	Gastric—Core	672	Exact match	UMIC	6.0	**
1	Cervical—Core	811	Partial match	Africa		**
1	Gastric—Enhanced	916	Exact match	UMIC	6.0	**
1	Prostate—Basic	1006	Exact match	UMIC	5.0	**
1	Prostate—Core	1403	Exact match	UMIC	5.0	**
1	Breast—Enhanced	1445	Exact match	Africa		**
1	Breast—Basic	1600	Partial match	Africa		**
1	Prostate—Enhanced	2881	Exact match	UMIC	1.0	**
2	Lymphoma—NHL—DLBCL—Basic	23	Exact match	Africa	4.0	***
2	Thyroid—Basic	50	Exact match	UMIC	4.0	**
2	Lymphoma—NHL—DLBCL—Core	450	Exact match	Africa	4.0	***
2	Oesophageal—Core	473	Partial match	UMIC	6.0	**
2	H&N—Core	814	Partial match	UMIC	6.0	**
2	Lymphoma—HL—Enhanced	893	Partial match	LMIC	5.5	**
2	Lung—NSCLC—Enhanced	999	Partial match	UMIC	5.0	**
2	Lung—SCLC—Enhanced	3065	Partial match	UMIC	4.0	**
2	Pancreatic—Enhanced	3609	Partial match	UMIC	5.0	**
2	Lung—Mesothelioma—Core	3808	Exact match	UMIC	5.0	**
2	Leukaemia—CML—Core	4265	Partial match	UMIC	5	**
2	Renal cell carcinoma—Enhanced	4537	Exact match	UMIC	5.0	**
2	Brain—Glioma—Core	4584	Partial match	UMIC	6.0	**
2	Brain—Glioma—Enhanced	4584	Partial match	UMIC	6.0	**
2	Skin—Melanoma—Enhanced	6431	Partial match	UMIC	5.0	**
2	Multiple myeloma—Enhanced	10 714	Partial match	UMIC	5.0	**
2	Lung—Mesothelioma—Enhanced	16 523	Exact match	UMIC	5.0	**
2	Oesophageal—Enhanced	17 922	Exact match	UMIC	5.0	**
2	Ovarian—Enhanced	43 708	Partial match	UMIC	5.5	**

ALL, acute lymphoblastic leukaemia; CML, chronic myeloid leukaemia; DALY, disability-adjusted life year; DLBCL, diffuse large B-cell lymphoma; HL, Hodgkin’s lymphoma; H&N, head and neck; LMIC, low- and middle-income country; NHL, non-Hodgkin’s lymphoma; NSCLC, non-small cell lung cancer; SCLC, small cell lung cancer; UMIC, upper-middle-income country; USD, US dollar.

### Elicited CERs

In round 2, the missing 98 CERs were elicited from the cancer experts. We received responses for 83 CERs (table 4). Those without response were for cancers that had no recent local incident cases, or those where there was no treatment assigned to a specific package (eg, a cancer was deemed by experts to only have a basic and enhanced package, but no core package) (n=15). Values for each CER were assigned in table 2.

**Table 4:** Elicited cost-effectiveness ratios

Cancer and level	CER		
Oesophageal—Basic	417	Lymphoma—NHL—DLBCL—Enhanced	1668
Skin—Melanoma—Basic	417	Ovarian—Core	1668
Adrenal tumours—Basic	417	Adrenal tumours—Core	1668
Anus—Enhanced	417	Brain—Brain tumours—Core	1668
Uterine—Enhanced*	417	Uterine—Core*	1668
Kaposi sarcoma—Basic	417	Kaposi sarcoma—Enhanced	1668
Neuroblastoma—Core	417	Leukaemia—ALL—Basic	1668
Penile—Basic	417	Leukaemia—AML—Core	1668
Penile—Enhanced	417	Leukaemia—AML—Enhanced	1668
Skin—Non-melanoma—Basic	417	Leukaemia—CLL—Basic	1668
Vulva/Vagina—Basic	417	Neuroblastoma—Enhanced	1668
Lung—NSCLC—Core	834	Renal pelvis carcinoma—Basic*	1668
Lymphoma—HL—Basic	834	Renal pelvis carcinoma—Enhanced*	1668
Lymphoma—HL—Core	834	Vulva/Vagina—Core	1668
Multiple myeloma—Basic	834	H&N—Enhanced	2502
Ovarian—Basic	834	Leukaemia—CML—Enhanced	2502
Renal cell carcinoma—Basic	834	Pancreatic—Basic	2502
Skin—Melanoma—Core	834	Pancreatic—Core	2502
Thyroid—Core	834	Thyroid—Enhanced	2502
Anus—Basic	834	Adrenal tumours—Enhanced	2502
Anus—Core	834	Bone—Enhanced	2502
Bone—Basic	834	Brain—Brain tumours—Enhanced	2502
Bone—Core	834	Leukaemia—ALL—Core	2502
Brain—Brain tumours—Basic	834	Leukaemia—ALL—Enhanced	2502
Uterine—Basic*	834	Leukaemia—AML—Basic	2502
Germ cell tumours—Basic	834	Leukaemia—CLL—Core	2502
Germ cell tumours—Core	834	Leukaemia—CLL—Enhanced	2502
Gestational/placenta—Basic	834	Skin—Non-melanoma—Enhanced	2502
Gestational/placenta—Core	834	Vulva/vagina—Enhanced	2502
Kaposi sarcoma—Core	834	Neuroendocrine tumours—Basic	2502
Lymphoma—NHL—T-cell—Basic	834	Neuroendocrine tumours—Core	2502
Neuroblastoma—Basic	834	Neuroendocrine tumours—Enhanced	2502
Penile—Core	834	Soft tissue sarcoma—Enhanced	2502
Renal pelvis carcinoma—Core*	834	Multiple myeloma—Core	–
Skin—Non-melanoma—Core	834	Renal cell carcinoma—Core	–
Soft tissue sarcoma—Basic	834	Germ cell tumours—Enhanced	–
Soft tissue sarcoma—Core	834	Gestational/Placenta—Enhanced	–
Brain—Glioma—Basic	1668	GIST—Basic	–
H&N—Basic	1668	GIST—Core	–
Leukaemia—CML—Basic	1668	GIST—Enhanced	–
Lung—Mesothelioma—Basic	1668	Lymphoma—NHL—T-cell—Core	–
Lung—NSCLC—Basic	1668	Lymphoma—NHL—T-cell—Enhanced	–
Lung—SCLC—Basic	1668	Thymoma/thymic carcinoma*—Basic	–
Lung—SCLC—Core	1668	Thymoma/thymic carcinoma*—Core	–
		Thymoma/thymic carcinoma*—Enhanced	–

‘–’ denotes either no incident cases were reported, or no treatment was included in this package.

* Each of the following includes two cancers for which CERs were sought: thymoma/thymic carcinoma (n=2), uterine (corpus uteri+endometrial) (n=2), renal pelvis (renal pelvis carcinoma+urothelial (n=2).

ALL, acute lymphoblastic leukaemia; AML, acute myeloid leukaemia; CER, cost-effectiveness ratio; CML, chronic myeloid leukaemia; DALY, disability-adjusted life year; DLBCL, diffuse large B-cell lymphoma; GIST, gastrointestinal stromal tumour; HL, Hodgkin’s lymphoma; H&N, head and neck; NHL, non-Hodgkin’s lymphoma; NSCLC, non-small cell lung cancer; SCLC, small cell lung cancer.

## Discussion

Our research sought CERs for 148 cancer packages for 49 cancers. We found 39 CERs in the published literature and elicited 83 CERs from the cancer experts. More exact matches to the full package of services were found in common cancers, whereas more partial matches focused on specific drugs were found in less common cancers. Ratios from the published literature ranged from 23 USD/DALY for the diffuse large B-cell lymphoma basic package to 43 708 USD/DALY for the ovarian enhanced package, after adjusting for PPP. Only two CERs were assigned three stars, with exact matches from studies from the African context; the remaining CERs were assigned two stars. Expert-elicited CERs included 11 packages which were considered very cost-effective, 19 which were considered not cost-effective and the remainder were potentially cost-effective. Experts identified proportionately more interventions which they considered very cost-effective, and fewer which they considered not cost-effective, compared with the published literature.

We estimate that the analytical time to complete the cost-effectiveness assessment was 3–4 months for a team of eight people working part-time. Access to the Tufts registry’s pre-extracted data was critical to expediting the assessment, as were the cancer experts who participated in the elicitation of missing CERs. Conducting the assessment built local capacity in systematic reviewing and reviewing of CEAs.

The strengths of this study include that it is the first of its kind, focused on assessing many cancers at once to inform HBP design. It adds to the existing HBP literature by providing details on how and why assessment methods were selected, and how the cost-effectiveness assessment was conducted. Our detailed reporting of the expert elicitation of CERs is a particularly unique addition to the HBP literature.

Further enhancements could have been made to the approach. The PPP adjustment could have been strengthened by splitting tradable and non-tradable goods and adjusting the latter for PPP,^33^ or CERs could have been recalculated with local costs. The assessment team could have also conducted a quality review of studies that were not quality reviewed by the Tufts registry. However, in adapting to time and capacity constraints, these additions were not possible.

Overall, there are several limitations to this work. These mostly stem from the fact that the gold standard of estimating cost-effectiveness would have been to conduct de novo analyses for all 148 interventions. Given that a single CEA would take about 1 year, this would be impossible and therefore, methods had to be adapted accordingly. The existing cost-effectiveness evidence reviewed was only available from the literature for about 25% of interventions, so the remainder were estimated using expert elicitation. While the experts were well prepared to provide inputs on CERs, the estimates provided are not precise and could include experts’ personal biases of what they consider to be cost-effective. The expert elicitation method presented here is novel and may benefit from further design refinement. There is overall uncertainty of the results, which stems from transferring evidence from one jurisdiction to another, lack of availability of relevant literature and reliance on expert opinion. Further elaboration of improvements for specific methodological limitations can be found in the recommendations in the following sections.

### Recommendations for producers and reviewers of economic evaluations

Reporting of cost-effectiveness analyses could be refined for use in HBPs. In Rwanda, the cancer experts were focused on curative, early-stage treatment which is uncommon in the CEA literature. Packages of care that were reported often covered stages I–IV, and recalculating CERs for a package of stages I–II or I–III was not possible. Study authors could consider reporting the costs and effects of each stage and groups of stages separately. Additionally, reporting units of costs and health effects in studies was inconsistent. Ideally, clearer reporting of costs and QALYs or DALYs per patient per year, size and definition of the study population, and total QALYs or DALYs averted per year would be consistently reported to enable accurate recalculations. There is also an obvious need for more CEAs to be done in LMICs, on LMIC priority topics.

Improvements could also be made to the Tufts database to avoid going back to original studies. The reporting of interventions was sometimes unclear. Analysts would benefit from more comprehensive intervention descriptions that include screening type, treatment method, drug(s) delivered and the line of treatment (first line, second line) for cancer. Furthermore, the reporting of *incremental* CERs versus ACERs was inconsistent and should always be clarified.

Ultimately, while cancer is a well-studied topic in other countries, transferring CEAs from various jurisdictions for HBP design is challenging and uncertain. Existing literature is disproportionately focused on a small subset of cancers, so methods had to be adapted to respond to the dearth of data for other cancers. The uncertainty of this approach creates a risk of suboptimal decision-making, which should be clearly communicated in appraisal proceedings.

### Recommendations for healthcare decision makers

Our findings demonstrate that any review of existing cost-effectiveness evidence should be conducted considering transferability to the local context. This at least includes geographic relevance, relevance of the intervention and comparator, and quality of the study. Additional transferability factors could be considered and are well documented in the literature on the transferability of economic evidence. If cost-effectiveness evidence is unavailable entirely, policy makers and analysts should carefully consider whether expert elicitation is appropriate for the interventions being reviewed, or if more research (such as de novo CEA) needs to be done to inform a decision. Selection and conduct of the methods in this paper reflect the number of interventions being reviewed (148), the topic (cancer) and local acceptability of methods (literature review with expert opinion).

## Conclusion

This cost-effectiveness assessment reviewed 49 cancers. It demonstrates the feasibility of combining rapid review with expert opinion to obtain CER estimates which were used to inform the prioritisation of a cancer package in Rwanda. Our study is the first of its kind to assess the cost-effectiveness of so many cancers at once for the purpose of HBP design. Moreover, it is the first HBP study to report methods of expert elicitation of CERs in detail, which should improve its future replicability.

## Supplementary material

10.1136/bmjph-2025-003718 online supplemental appendix 1

## Data Availability

Data are available upon reasonable request.
