# Supplementary material for: Cost-effectiveness of cancer interventions in Rwanda: literature review and expert elicitation for health benefits package design
Source: BMJ Public Health. 2026 Mar 5;4(1):e003718. doi: 10.1136/bmjph-2025-003718 (PMC12970044; doi:10.1136/bmjph-2025-003718)
Supplement: online supplemental appendix 1 [file bmjph-4-1-s001.docx]

Appendix 1: Incidence of Cancer in Rwanda

Cancers highlighted in grey are those which were assessed in assessment one (where colon and rectal cancers are counted as two cancers).

| No | Cancer | Cases (2019) |
| --- | --- | --- |
| 1 | Breast cancer | 552 |
| 2 | Cervical cancer | 535 |
| 3 | Prostate cancer | 401 |
| 4 | Stomach cancer | 362 |
| 5 | Liver cancer | 258 |
| 6 | Colon and rectum cancer | 182 |
| 7 | Adult Chronic myelogenous leukemia | 148 |
| 8 | Adult non-hodgkins lymphoma - DLBCL | 148 |
| 9 | Sarcoma | 120 |
| 10 | Head and Neck | 97 |
| 11 | Kaposi Sarcoma | 60 |
| 12 | Penile | 51 |
| 13 | Oesophageal | 49 |
| 14 | Hodgkin Lymphoma | 47 |
| 15 | Wilms Tumor | 40 |
| 16 | Acute Lymphoblastic Leukemia | 39 |
| 17 | Retinoblastoma | 39 |
| 18 | Bladder | 38 |
| 19 | Gestational trophoblastic neoplasia (GTN) | 35 |
| 20 | Ovarian Bep | 30 |

Appendix 2: Cancers assessed

| Round 1 | | Round 2 | | | |
| --- | --- | --- | --- | --- | --- |
| 1 | Cervical | 1 | Adrenal tumors | 21 | Lymphoma - NHL - T-cell |
| 2 | Breast | 2 | Anus | 22 | Multiple myeloma |
| 3 | Colon | 3 | Bone | 23 | Neuroblastoma |
| 4 | Rectal | 4 | Brain - brain tumors | 24 | Neuroendocrine tumors |
| 5 | Liver | 5 | Brain – glioma | 25 | Ovarian |
| 6 | Gastric | 6 | Esophageal | 26 | Pancreatic |
| 7 | Prostate | 7 | Germ cell tumors | 27 | Penile |
| 8 | Retinoblastoma | 8 | Gestational | 28 | Renal cell carcinoma |
| 9 | Wilms | 9 | GIST | 29 | Renal pelvis carcinoma |
| 10 | Acute Lymphoblastic Leukemia | 10 | H & N | 30 | Skin – Melanoma |
|  |  | 11 | Kaposi sarcoma | 31 | Skin - Non-melanoma |
|  |  | 12 | Leukemia – ALL | 32 | Soft tissue sarcoma |
|  |  | 13 | Leukemia – AML | 33 | thymic carcinoma |
|  |  | 14 | Leukemia – CLL | 34 | Thymoma |
|  |  | 15 | Leukemia – CML | 35 | Thyroid |
|  |  | 16 | Lung – Mesothelioma | 36 | Urothelial |
|  |  | 17 | Lung – NSCLC | 37 | Uterine - corpus uteri |
|  |  | 18 | Lung – SCLC | 38 | Uterine - endometrial |
|  |  | 19 | Lymphoma – HL | 39 | Vulva/vagina |
|  |  | 20 | Lymphoma - NHL – DLBCL |  |  |

Appendix 3: Search strategies

Round 1

Cancer OR Neoplasm OR Oncology OR Malignant OR Malignancy OR Metastatic OR Metastasis OR Tumor OR Tumour OR Nephroblastoma OR Wilms' tumor OR Wilms' tumour OR Lymphoma OR Leukemia OR Leukaemia OR Breast cancer OR Kaposi’s sarcoma OR Prostate cancer OR Colorectal cancer OR Cervical cancer OR Liver cancer OR Gastric cancer OR Eye cancer OR Osteosarcoma OR Malignant gestational trophoblastic disease OR Head and neck cancer OR Abiraterone OR Anastrozole OR Bevacizumab OR Bleomycin OR Calcium folinate (leucovorin) OR Capecitabine OR Carboplatin OR Cisplatin OR Cyclophosphamide OR Cyclosporine OR Docetaxel OR Doxorubicin OR Fluorouracil OR Folinic acid OR Goserelin OR Hydroxycarbamide Tamoxifen Citrate OR Ifosfamide OR Imatinib OR Irinotecan OR L-asparginase OR Letrozole OR Melphalan OR Mercaptopurine OR Methotrexate OR Mycophenolate OR Oxaliplatin OR Paclitaxel OR Rituximab OR Sorafenib OR Trastuzumab OR Vincristine OR Zoledronate OR Zoledronic Acid

Round 2

Cancers

brain cancer OR brain metastases OR brain tumor OR brain tumour OR medulloblastoma OR head and neck OR oral cavity cancer OR hypopharynx OR laryngeal OR oropharyngeal OR nasopharyngeal OR nasal cavity OR sinus cancer OR squamous cell carcinoma OR thyroid cancer OR thyroid carcinoma OR lung cancer OR small cell lung cancer OR non-small cell lung cancer OR SCLC OR NSCLC OR soft tissue sarcoma OR skin melanoma OR skin cancer OR anal cancer OR epidermoid cancer OR esophageal cancer OR oesophageal cancer OR pancreatic cancer OR adenocarcinoma OR sarcoma OR gastrointestinal stromal tumour OR gastrointestinal stromal tumor OR GIST OR neuroendocrine tumour OR neuroendocrine tumor OR ureter cancer OR bladder cancer OR testicular cancer OR penile cancer OR ovarian cancer OR vulvar cancer OR vulva cancer OR uterine cancer OR endometrial carcinoma OR Gestational trophoblastic disease OR vaginal cancer OR gallbladder cancer OR choriocarcinoma OR Hodgkin lymphoma OR Hodgkin disease OR non-Hodgkin lymphoma OR non-Hodgkin disease OR diffuse large b cell lymphoma OR Burkitt lymphoma OR leukemia OR leukaemia OR chronic myeloid leukemia OR chronic myeloid leukaemia OR CML OR CLL OR chronic lymphocytic leukaemia OR chronic lymphocytic leukemia OR multiple myeloma OR lymphoma OR adrenal cancer OR Adrenocortical carcinoma OR bone cancer OR osteosarcoma OR ewing sarcoma OR yolk sac OR mesothelioma OR gestational trophoblastic neoplasia

Drugs

5-FU OR 5FU OR Actinomycin OR Adriamycin OR Afatinib OR Alectinib OR Anastrazole OR Bendamustine  OR Bevacizumab OR Bleomycin OR Bortezomib OR Capecitabine OR Carboplatin OR Chlorambucil OR CHOP OR Cisplatin OR Crizotinib OR CyBorD OR Cyclophosphamide OR Cytarabine OR Dabrafenib  OR Dacarbazine OR Dactinomycin OR Dasatinib OR Denosumab OR Dexamethasone OR Docetaxel OR Doxorubicin OR EP OR Epirubicin OR Erlotinib OR Etoposide OR Exemestane OR Filgrastim OR Fludarabine OR FOLFIRINOX OR Gemcitabine OR GEMOX OR Hydroxyurea OR Ifosfamide OR Imatinib OR Irinotecan OR Lenalidomide OR Letrozole OR Leucovorin OR Mesna OR Methotrexate OR Mitomycin OR Nivolumab OR Oxaliplatin OR Paclitaxel OR Pembrolizumab OR Pemetrexed OR Prednisone OR R-CHOP OR Rituximab OR Temozolomide OR Topotecan OR Trametinib OR Vinblastine OR vincristine OR Vinorelbine

Appendix 4: Overview of cancer experts

| **Type of expertise** | **Number of experts** |
| --- | --- |
| NCD division manager | 1 |
| Medical oncologist | 2 |
| Radiation oncologist | 1 |
| Pathologist | 1 |
| Radiologist | 1 |
| Oncology pharmacist | 1 |
| Surgical oncologist | 1 |
| Oncology nurse | 2 |
| Cancer director – Rwanda biomedical center | 1 |
| NCD director – Rwanda biomedical center | 1 |

Appendix 5: Studies included

The studies below are those which were included in the final cancer recommendation. Either the intervention or comparator is bolded for each – the bolded one is the intervention we included in assessment.

| Round | Cancer and level | Author & Year | Title | Country | Intervention | Comparator |
| --- | --- | --- | --- | --- | --- | --- |
| 1 | Breast - Basic | Zelle 2005 | Costs, effects and cost-effectiveness of breast cancer control in Ghana | Ghana | **Treatment of breast cancer, stages I-IV** | None |
| 1 | Breast - Core | Zelle 2005 | Costs, effects and cost-effectiveness of breast cancer control in Ghana | Ghana | **Biennial clinical breast examination (CBE) screening + Treatment of breast cancer, stages I-IV** | None |
| 1 | Breast - Enhanced | Zelle 2005 | Costs, effects and cost-effectiveness of breast cancer control in Ghana | Ghana | **Biennial mammography screening + Treatment of breast cancer, stages I-IV** | None |
| 1 | Cervical - Prevention | Jit 2005 | Cost-effectiveness of female human papillomavirus vaccination in 179 countries: a PRIME modelling study | Rwanda | **Human papillomavirus (HPV) vaccination** | None |
| 1 | Cervical - Basic | Ginsberg 2012 | Cost effectiveness of strategies to combat breast, cervical, and colorectal cancer in sub-Saharan Africa and South East Asia: mathematical modelling study | SSA | **Visual inspection for cervical cancer with acetic acid (VIA) at ages 35, 40, and 45 (with lesion removal) + cancer treatment** | None |
| 1 | Cervical - Basic | Kim 2006 | Packaging health services when resources are limited: the example of a cervical cancer screening visit | SSA | **Visual inspection using acetic acid for cervical cancer** | None |
| 1 | Cervical - Core | Kim 2006 | Packaging health services when resources are limited: the example of a cervical cancer screening visit | SSA | **HPV DNA test for cervical cancer** | None |
| 1 | Cervical - Enhanced | Ginsberg 2012 | Cost effectiveness of strategies to combat breast, cervical, and colorectal cancer in sub-Saharan Africa and South East Asia: mathematical modelling study | SSA | **Smear test for cervical cancer detection every 5 years, ages 20-65 + HPV vaccine from age 12 + cancer treatment** | None |
| 1 | ALL - Basic | Fuentes-Alabi 2018 | The cost and cost-effectiveness of childhood cancer treatment in El Salvador, Central America: A report from the Childhood Cancer 2030 Network | El Salvador | **Cancer treatment at the Hospital Nacional de Ninos Benjamin Bloom (HNNBB) in San Salvador** | None |
| 1 | Retinoblastoma - Basic | Renner 2018 | Evidence From Ghana Indicates That Childhood Cancer Treatment in Sub-Saharan Africa Is Very Cost Effective: A Report From the Childhood Cancer 2030 Network | Ghana | **Pediatric oncology treatment center** | None |
| 1 | Wilm's Tumour - Basic | Renner 2018 | Evidence From Ghana Indicates That Childhood Cancer Treatment in Sub-Saharan Africa Is Very Cost Effective: A Report From the Childhood Cancer 2030 Network | Ghana | **Pediatric oncology treatment center** | None |
| 1 | Wilm's Tumour - Basic | Fuentes-Alabi 2018 | The cost and cost-effectiveness of childhood cancer treatment in El Salvador, Central America: A report from the Childhood Cancer 2030 Network | El Salvador | **Cancer treatment at the Hospital Nacional de Ninos Benjamin Bloom (HNNBB) in San Salvador** | None |
| 1 | Colorectal - Core | Ginsberg 2012 | Cost effectiveness of strategies to combat breast, cervical, and colorectal cancer in sub-Saharan Africa and South East Asia: mathematical modelling study | SSA | **Colonoscopy at age 50 (with surgical removal of polyps)+ cancer treatment** | None |
| 1 | Colorectal - Enhanced | Ginsberg 2012 | Cost effectiveness of strategies to combat breast, cervical, and colorectal cancer in sub-Saharan Africa and South East Asia: mathematical modelling study | SSA | **Colonoscopy screening every 10 years + cancer treatment** | None |
| 1 | Gastric - Core | Zhang 2019 | Adjuvant Chemoradiotherapy for Gastric Cancer: Efficacy and Cost-Effectiveness Analysis | China | **adjuvant chemotherapy for gastric cancer** | None |
| 1 | Gastric - Enhanced | Zhang 2019 | Adjuvant Chemoradiotherapy for Gastric Cancer: Efficacy and Cost-Effectiveness Analysis | China | **adjuvant chemoradiotherapy for gastric cancer** | None |
| 1 | Gastric - Basic | Zhang 2019 | Adjuvant Chemoradiotherapy for Gastric Cancer: Efficacy and Cost-Effectiveness Analysis | China | **adjuvant chemotherapy for gastric cancer** | None |
| 1 | Prostate - Basic | Zhang 2016 | Addition of docetaxel and/or zoledronic acid to standard of care for hormone-naive prostate cancer: a cost-effectiveness analysis | China | **Docetaxel + standard of care** | Standard/Usual Care |
| 1 | Prostate - Core | Zhang 2016 | Addition of docetaxel and/or zoledronic acid to standard of care for hormone-naive prostate cancer: a cost-effectiveness analysis | China | **Docetaxel + standard of care** | Standard/Usual Care |
| 1 | Prostate - Enhanced | Aguiar 2018 | Cost-effectiveness analysis of abiraterone, docetaxel or placebo plus androgen deprivation therapy for hormone-sensitive advanced prostate cancer | Brazil | **abiraterone + androgen deprivation therapy** | Standard/Usual Care- androgen deprivation therapy |
| 2 | Brain - glioma - Enhanced | Wu 2012 | Subgroup economic analysis for glioblastoma in a health resource-limited setting | China | **Temozolomide and radiotherapy (TMZ + RT)** | Radiotherapy |
| 2 | Brain - glioma - Core | Wu 2012 | Subgroup economic analysis for glioblastoma in a health resource-limited setting | China | **Temozolomide and radiotherapy (TMZ + RT)** | Radiotherapy |
| 2 | Esophageal - Enhanced | Zhang 2020 | Cost-effectiveness analysis of nivolumab in the second-line treatment for advanced esophageal squamous cell carcinoma | China | **Nivolumab** | Standard/Usual Care- chemotherapy (paclitaxel and/or - unclear - docetaxel) |
| 2 | Esophageal - Core | Zhan 2019 | Cost-effectiveness analysis of neoadjuvant chemoradiotherapy followed by surgery versus surgery alone for locally advanced esophageal squamous cell carcinoma based on the NEOCRTEC5010 trial | China | **Neoadjuvant chemoradiotherapy (vinorelbine + cisplatin) + surgery** | Standard/Usual Care- surgery alone |
| 2 | H & N - Core | Yang 2020 | Real-World Cost-Effectiveness Analysis of Gemcitabine and Cisplatin Compared to Docetaxel and Cisplatin Plus Fluorouracil Induction Chemotherapy in Locoregionally Advanced Nasopharyngeal Carcinoma | China | Gemcitabine + cisplatin --> cisplatin + intensity modulated radiotherapy | **Docetaxel + fluorouracil + cisplatin + radiotherapy** |
| 2 | Leukemia - CML - Core | Li 2017 | Cost Effectiveness of Imatinib, Dasatinib, and Nilotinib as First-Line Treatment for Chronic-Phase Chronic Myeloid Leukemia in China | China | Dasatinib first | **Imatinib first** |
| 2 | Lung - NSCLC - Enhanced | Limwattananon 2018 | Cost-effectiveness analysis of policy options on first-line treatments for advanced, non-small cell lung cancer in Thailand | Thailand | EGFR test; Afatinib M+/Platin M- | **Carboplatin + paclitaxel (and other platinum doublets)** |
| 2 | Lung - Mesothelioma - Enhanced | Zhan 2017 | Cost-effectiveness analysis of additional bevacizumab to pemetrexed plus cisplatin for malignant pleural mesothelioma based on the MAPS trial | China | **Pemetrexed/cisplatin plus bevacizumab** | Standard/Usual Care- Pemetrexed/cisplatin |
| 2 | Lung - Mesothelioma - Core | Zhan 2017 | Cost-effectiveness analysis of additional bevacizumab to pemetrexed plus cisplatin for malignant pleural mesothelioma based on the MAPS trial | China | Pemetrexed/cisplatin plus bevacizumab | **Standard/Usual Care- Pemetrexed/cisplatin** |
| 2 | Lung - SCLC - Enhanced | Zhou 2017 | Cost-effectiveness analysis of sensitive relapsed small-cell lung cancer based on JCOG0605 trial | China | **Cisplatin, etoposide, irinotecan** | Standard/Usual Care- Topotecan |
| 2 | Lymphoma - HL - Enhanced | Hatam 2015 | Cost-Utility Analysis of IEV Drug Regimen Versus ESHAP Drug Regimen for the Patients With Relapsed and Refractory Hodgkin and Non-Hodgkin's Lymphoma in Iran | Iran | **Ifosfamide, epirubicin and etoposide (IEV Treatment)** | etoposide, methylprednisolone, high-dose cytarabine, and cisplatin (ESHAP Treatment) |
| 2 | Lymphoma - NHL - DLBCL - Core | Painschab 2021 | Comparison of best supportive care, CHOP, or R-CHOP for treatment of diffuse large B-cell lymphoma in Malawi: a cost-effectiveness analysis. | Malawi | **R-CHOP (individual patient)** | CHOP (individual patient) |
| 2 | Lymphoma - NHL - DLBCL - Basic | Painschab 2021 | Comparison of best supportive care, CHOP, or R-CHOP for treatment of diffuse large B-cell lymphoma in Malawi: a cost-effectiveness analysis. | Malawi | CHOP (individual patient) | **best supportive care, palliative treatment without chemotherapy (individual patient)** |
| 2 | Multiple myeloma - Enhanced | Cai 2019 | Cost-effectiveness analysis on binary/triple therapy on the basis of ixazomib or bortezomib for refractory or relapsed multiple myeloma | China | **Bortezomib, Thalidomide, and Dexamethasone** | Bortezomib and Dexamethasone |
| 2 | Ovarian - Enhanced | Luealon 2016 | Cost Effectiveness Analysis of Different Management Strategies between Best Supportive Care and Second-line Chemotherapy for Platinum-resistant or Refractory Ovarian Cancer | Thailand | **Gemcitabine + BSC** | Standard/Usual Care- Best supportive care |
| 2 | Pancreatic - Enhanced | Cui 2020 | Cost-effectiveness analysis of nab-paclitaxel plus gemcitabine versus folfirinox in the treatment of metastatic pancreatic cancer in china | China | Nab-paclitaxel + gemcitabine | **Standard/Usual Care- fluorouracil + leucovorin + irinotecan + oxaliplatin (folfirinox)** |
| 2 | Renal cell carcinoma - Enhanced | Chen 2019 | Cost-effectiveness Analysis of Pembrolizumab Plus Axitinib Versus Sunitinib in First-line Advanced Renal Cell Carcinoma in China | China | Pembrolizumab + axitinib | **Sunitinib** |
| 2 | Skin - Melanoma - Enhanced | Gao 2021 | Cost-Effectiveness Analysis of Dabrafenib Plus Trametinib and Vemurafenib as First-Line Treatment in Patients with BRAF V600 Mutation-Positive Unresectable or Metastatic Melanoma in China. | China | **Dabrafenib** | vemurafenib |
| 2 | Thyroid - Basic | Corso 2014 | Total thyroidectomy versus hemithyroidectomy for patients with follicular neoplasm. A cost-utility analysis | Colombia | Partial thyroidectomy | **Total thyroidectomy** |
| Note: the **bolded** intervention or comparator is what was used to calculate the ACER. | | | | | | |
